# Bispectral Index and Surgical Space Conditions in Day Surgery Benign Gynecological Laparoscopies: A Double-Blinded Randomized Clinical Trial

**DOI:** 10.1155/anrp/4558323

**Published:** 2025-02-23

**Authors:** Elena Crescioli, Peter Søndergaard Thyrrestrup, Thale Almås

**Affiliations:** ^1^Department of Anesthesia and Intensive Care, Aalborg University Hospital, Aalborg, Denmark; ^2^Department of Clinical Medicine, Aalborg University, Aalborg, Denmark; ^3^Department of Anesthesia and Intensive Care, Odense University Hospital, Odense, Denmark

## Abstract

**Background:** Ambulatory surgery is increased in numbers, healthcare costs are reduced, and patient safety is high when patient characteristics and type of surgery are properly evaluated. To maintain efficiency and patient safety, anesthesia and surgery must be optimized. The bispectral index (BIS) is a widely used and simplified measure of the depth of anesthesia and may provide a more stable anesthesia and avoid insufficient levels of anesthesia. We investigated whether BIS–guided anesthesia could increase the frequency of excellent overview of the surgical field.

**Methods:** This is a single-center double-blinded randomized clinical trial. We enrolled patients undergoing gynecologic laparoscopies on a benign indication from April 2019 to March 2021. Using closed envelopes, patients were randomized to receive either a BIS–assisted anesthesia, or to receive anesthesia without BIS monitoring (i.e., control group). Surgeons graded their surgical field overview using a four-grade numerical scale at three predefined time points. The primary outcome was the proportion of patients graded with an excellent surgical overview.

**Results:** We included 160 women: 80 patients were randomized to the BIS group and 80 to the control group. There was no statistically significant difference between groups in excellent surgical overview at any time point. Among secondary outcomes, a lower remifentanil consumption in the control group was found in comparison with the BIS group.

**Conclusions:** This trial found no clinically relevant differences regarding surgical overview quality during gynecological laparoscopic surgery when using BIS to titrate anesthesia. To our knowledge, this is the first study investigating this aspect.

**Trial Registration**: ClinicalTrials.gov identifier: NCT03911544

## 1. Introduction

In the last decades, day surgery has expanded and become a recognized specialty [1]. The fast tracking succeeds when the patient spends less time in the hospital, reducing the costs without compromising the quality of care. In this regard, the anesthesiologist plays a central role both in the preoperative evaluation and in taking care of the patient throughout the perioperative period. Furthermore, the anesthesiologist's goal is to provide the best anesthesia to ease surgeon's work and reduce the complication rate [2]. Several clinical trials have investigated the impact of moderate versus deep neuromuscular block (NMB) on surgical space conditions in laparoscopies using subjective rating scores [3–6]. The mentioned scores are not validated but have been widely used in these trials as surrogates of operator satisfaction and consequently surgical conditions.

The bispectral index (BIS) processes the electroencephalogram in near real time and provides an empirically derived scale, which reflects the patient's level of consciousness [7]. Since its release in 1996, the BIS has been increasingly used by anesthesia providers, who have praised its benefits in titrating hypnotics more accurately, reducing the risk of awareness in high-risk patients and enhancing patient´s recovery, as described in a recent Cochrane review [8]. At present, no study has investigated whether BIS–assisted anesthesia would improve laparoscopic surgical conditions.

To explore this, we conducted a prospective, randomized clinical trial, in which patients scheduled for a day surgery gynecological laparoscopy were randomized to either BIS–assisted total intravenous anesthesia (TIVA), or TIVA guided by clinical signs. We hypothesized that TIVA assisted by BIS monitoring would increase the frequency of excellent operating conditions at any time point.

## 2. Methods

### 2.1. Trial Design and Population

The present trial was a single-center, randomized, double-blinded clinical trial. Upon a written informed consent, we enrolled patients scheduled for a day surgery gynecological laparoscopy between April 2019 and March 2021 at Aalborg University Hospital, Denmark. Patients were enrolled in the preoperative evaluation by the anesthesiologists of the department. The inclusion was paused twice due to the COVID-19 pandemic outbreak and the consequent suspension of day surgery activities. Inclusion criteria were as follows: 18 years of age or older, American Society of Anesthesiology (ASA) physical status of 1 or 2, scheduled laparoscopic gynecological surgery, and legally competent. Exclusion criteria were as follows: renal or hepatic disease, laparoscopic surgery for cancer, and relevant allergies towards anesthetics.

The trial was approved by the Ethical Committee of Region Nordjylland, Aalborg, Denmark (18^th^ of March 2019, N-20190006). This report was prepared in accordance with the Consolidated Standards of Reporting Trials (CONSORT) [9] (checklist is presented in the Electronic Supporting Material (ESM)).

### 2.2. Randomization and Intervention

Patients were randomized 1:1 to either the BIS group or the control (CNTRL) group before the scheduled surgery. Randomization was performed in clusters of 10 (i.e., 5 CNTRL and 5 intervention) through closed envelopes prepared by the study sponsor. The randomization was performed by the administrative staff with no insight into the patients' charts, characteristics, or type of surgery.

In the BIS group, the depth of anesthesia was adjusted with the help of BIS monitoring. In the CNTRL group, the depth of anesthesia was guided by clinical signs. Patients, surgeons, and postoperative care nurses were blinded to the group allocation. The senior anesthesiologist in charge and anesthetist nurses were unblinded to the intervention and strictly observed the anesthesia protocol as explained in the next section. The protocol was kept unchanged throughout the trial.

### 2.3. Perioperative Protocol

The patients did not receive premedication. The standard monitoring equipment consisted of three lead electrocardiograms, noninvasive blood pressure, pulse oximetry, and BIS (BIS VISTA 3.26 SW). In the CNTRL group, the BIS monitor was covered before the induction of anesthesia. A single peripheral intravenous line was inserted and connected to an infusion of Ringer acetate solution. While the patient was exposed to a fraction of inspired oxygen of 100% through a facemask, anesthesia was induced with remifentanil 0.8 μg/kg/min and propofol 2 mg/kg. When the patient lost consciousness, the ventilation was controlled manually. An orotracheal intubation was performed after no less than 3 min and making sure that the patient had received at least 3 μg/kg remifentanil. Anesthesia was maintained with propofol 4–6 mg/kg/h and remifentanil 0.5–0.9 μg/kg/min. Controlled mechanical ventilation was performed with a tidal volume of 6–7 mL/kg and positive end–expiratory pressure (PEEP) of 5 cmH_2_O.

In the CNTRL group, anesthesia was adjusted according to clinical signs. If clinical signs of poor anesthesia were present (e.g., an increase in the arterial pressure and/or heart rate, tearing, and profuse sweating), anesthesia was deepened by titrating the TIVA infusion rate. In the BIS group, anesthesia was adjusted to target BIS values between 40 and 60. In case of clinical signs of poor anesthesia, the course of action depended on BIS values. If BIS values were in the target range, the remifentanil infusion rate was increased by 0.2 μg/kg/min every 5 min. If BIS showed values over 60, anesthesia was deepened by increasing the propofol infusion rate. Finally, if BIS values were below 40, the propofol infusion was reduced by 0.5 mg/kg/h until BIS readings were in the target range. In both groups, a hypotension episode (e.g., reduced arterial pressure by more than 20% from the baseline) was treated by a fluid challenge (250 mL Ringer acetate solution), ephedrine 5 mg or phenylephrine 0.1 mg, eventually reducing TIVA infusion rate. Bradycardia was managed with bolus atropine 0.015 mg/kg. Other anesthesia-related issues and complications were handled according to the local guidelines. Patients received intravenous paracetamol 1 g and dexamethasone 4 mg after anesthesia induction, as well as ketorolac 30 mg, ondansetron 4 mg, and fentanyl 2 μg/kg 10–20 min before the end of anesthesia. In addition, bupivacaine 1–1.5 mg/kg was injected into the surgical incisions.

### 2.4. Surgery and Evaluation of Surgical Conditions

Surgeons entered the operating room after induction of anesthesia and were blinded to group allocation; they were not familiar with BIS monitoring and received no introduction to this device. The surgery began with intraperitoneal insufflation of carbon dioxide with a Veress needle at the umbilicus. The insufflation pressure was set and monitored at 12 mmHg, and, if possible, lowered to 8 mmHg, throughout the procedure. One or two additional ports were entered in the suprapubic area depending on the type of surgery. Surgery was performed as outlined by local recommendations. The surgeon in charge was asked to assess the exposure of the surgical field on a four-grade numerical scale, with the following scores: (1) excellent, (2) good but not optimal, (3) poor but acceptable, and (4) unacceptable and impossible to continue the operation [4, 9, 10]. A chart with the scale was shown to the surgeon at every assessment. The time points of assessments were at the establishment of the pneumoperitoneum, 10 min after, and just before the deflation of the pneumoperitoneum.

If the exposure of the surgical field was rated poor or unacceptable (Score 3 or 4), remifentanil infusion was increased by 0.2 μg/kg/min with 5 min intervals. If the infusion rate reached 0.9 μg/kg/min and the surgical field assessment was still 3 or 4, a bolus of mivacurium 0.2 mg/kg was administered, irrespective of the group. Patients receiving mivacurium were monitored with a train of four ratios. If NMB was needed, the propofol infusion rate in the CNTRL group was increased to a minimum of 5 mg/kg/h to reduce the risk of awareness. TThe infusion rate in the BIS group was determined by the BIS reading. After surgery, the extubated patient was transferred to the postanesthesia care unit (PACU). Further details about the monitoring in the PACU are described in the ESM.

### 2.5. Outcome Measures

The primary outcome was the proportion of the lowest surgical field score (i.e., excellent surgical condition) at three different time points. Secondary outcomes were as follows: administration of NMB (yes/no and dosage), perioperative consumption of anesthetics and narcotics (dosage), BIS values, events of postoperative nausea and vomiting in PACU, antiemetic administration in PACU (yes/no, and dosage), and analgesics administration in PACU (yes/no, and dosage).

### 2.6. Statistics

An a priori power analysis indicated a total sample size of 148 patients needed to be included in the study to reach adequate power to find an improvement in optimal surgical conditions from 40% to 62.5% in the BIS group (power of 80% and an *α* of 5%). We included 160 patients to cover potential patient withdrawals.

Baseline variables are reported as numbers and percentages for categorical variables and median and interquartile range (IQR) for continuous variables, as applicable. According to the CONSORT 2010 statement [11], group differences in baseline variables were not compared using significance testing.

We conducted the primary analyses in the intention-to-treat population, defined as the patients randomized excluding those for whom consent for the use of data was withdrawn or those who did not receive the surgery. We also performed secondary analyses of all outcomes in the per-protocol analysis. As we defined the primary outcome as the proportion of best surgical condition (i.e., a score equal to 1), we dichotomized the scale into either excellent surgical condition (i.e., a score equal to 1) or other than excellent (i.e., any score above 1). We tested the primary outcome at each time point using a *X*^2^-test. Due to the performance of the three tests, we applied the Bonferroni correction and considered the adjusted *p* value of 0.0167 as significant.

The categorical secondary outcomes were compared between the intervention and CNTRL groups using the *X*^2^-test. The continuous secondary outcomes were tested for normal distribution using standardized normal probability plots; if the outcome was not judged to follow a normal distribution, we used a log transformation and re-evaluated normality. An unpaired *t*-test or Wilcoxon rank-sum test was used as appropriate. A 5% significance level was used in all the analyses of the secondary outcomes. All analyses were conducted in STATA Version 16.1 (StataCorp. 2019, Stata Statistical Software: Release 16, College Station, TX: StataCorp LLC).

## 3. Results

**Figure 1:**
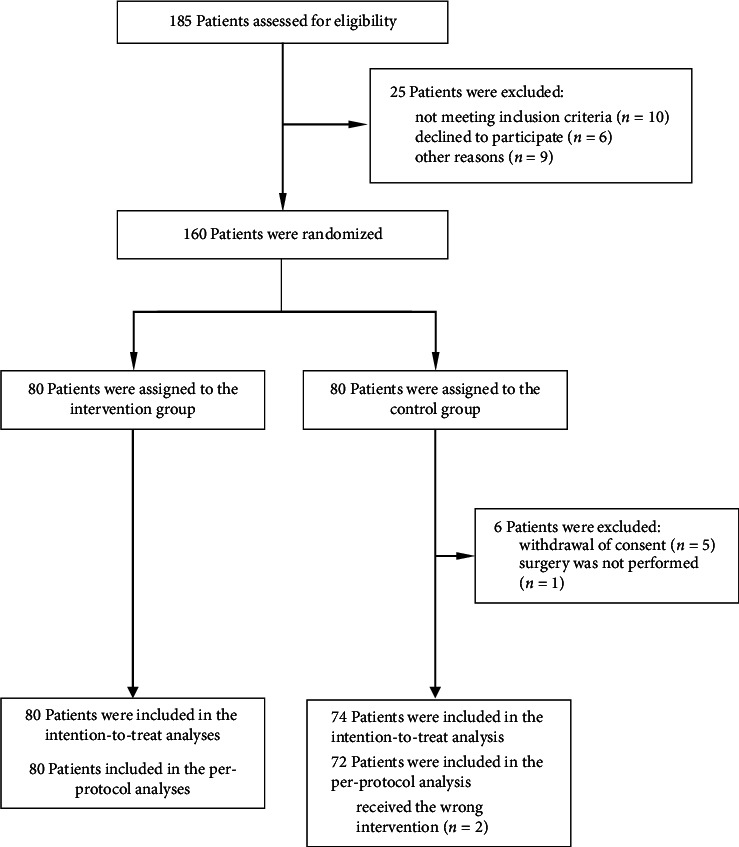
CONSORT flow diagram.

Between April 1, 2019, and March 11, 2021, 185 patients were assessed for eligibility and 160 were randomized. We included 154 patients in the final analysis: 80 in the BIS group B and 74 in the CNTRL group (Figure 1). Patient characteristics and perioperative details were similar in the two allocation groups (Table 1). In the BIS group, mean (SD) BIS values were 45 at pneumoperitoneum, 44.6 (8.4) after 10 min, and 43.7 (7.7) at deflation of pneumoperitoneum (Figure S1).

The analyses of the primary outcome are presented in Table 2: no statistically significant differences were detected between groups at any time point. Further details of the primary outcome assessments at the three time points are presented in the ESM (Figure S2) The secondary outcomes are shown in Table 3: we did not find any statistically significant difference except for a lower remifentanil consumption in the CNTRL group (*p*=0.005). The BIS group had more than twice as many patients reporting nausea in the PACU compared to the CNTRL group; however, only one patient in each group received antiemetic medicines, 10 mg primperan in the CNTRL group and 4 mg ondansetron in the BIS group. Numerical rating scale (NRS) scores recorded at three time points in PACU were also comparable, as presented in Table S1. We were not able to collect data on the continuous BIS values in both groups. The secondary analyses of all outcomes in the per-protocol population are reported in the ESM (Tables S2–S3).

## 4. Discussion

In this trial investigating the effects of a TIVA protocol guided by BIS versus clinical signs on surgical conditions in adult females undergoing benign gynecological laparoscopies, we found that BIS monitoring did not improve the surgical overview.

This is the first trial exploring the influence of BIS–assisted anesthesia on laparoscopic surgical conditions. Previous studies focussed primarily on the role of NMB [3–6, 10, 11]. However, a Danish cohort study described that almost one-third of more than 100.000 analyzed surgical procedures, including laparoscopy and tracheal intubation, which were performed without the use of NMB [13], and this was also the consolidated experience at our center. Therefore, we decided to investigate whether BIS–assisted anesthesia would play a role in improving the operating conditions without the routine use of NMB. The findings of our trial did not support the hypothesis that a more stable anesthesia provided by BIS monitoring would result in a better surgical overview during laparoscopic surgery. This was also reflected by a similar consumption of NMB in the two groups. Interestingly, we found a higher consumption of remifentanil in the BIS group in comparison to the CNTRL group. According to the study protocol, remifentanil was increased if the surgical overview after establishing pneumoperitoneum was judged poor or unacceptable. Since we did not find any differences in surgical field scores, except for the third time point, and no documentation for increased remifentanil administration was reported in our data collection, the finding is possibly of spurious nature and should be interpreted with caution. Furthermore, the propofol consumption was similar in both groups. In a systematic review and meta-analysis performed by Punjasawadwong, Phongchiewboon, and Bunchungmongkol for exploring the role of BIS in the anesthetic delivery, a significant effect of BIS in reducing the propofol consumption was found [14]. The systematic review was updated in 2019 [8], but these outcomes were not included. In our trial, the lack of propofol reduction in the BIS–guided anesthesia may be explained by the relatively short operative time, with a mean below 60 min in both groups, and by the fact that induction of anesthesia was weight-related in both groups regardless of BIS values. The need for additional opioids in the PACU was higher in the CNTRL group and consistent with NRS scores, with 41% of patients in the CNTRL group reporting a NRS over 4 at 30 min after arrival in the PACU versus 34% of patients in the BIS group. A higher rate of conversion to open surgery in the CNTRL group may explain some of the differences, which did not reach the statistical significance. This study investigated propofol and remifentanil as the preferred drugs in the ambulatory setup. Since we designed the study, remimazolam has gained interest as an alternative sedative in ambulatory anesthesia due to its promising pharmacokinetics advantages [15]. Different drugs may have different impacts on EEG measurements, and some studies have implied that BIS values may be higher in individuals anesthetised using remimazolam [16]. Until further data are provided BIS measurements must be interpreted with caution using remimazolam, and further research is needed to investigate the impact on surgical field scores when using remimazolam for laparoscopic procedures.

### 4.1. Strengths and Limitations

A strength of our trial is the well-predefined protocol registered at Clinicaltrials.gov before the enrollment of the first patient. Furthermore, the use of a pragmatic design allowed us to include the prespecified population in an acceptable amount of time, with minimal loss to follow-up. However, the enrollment was prolonged due to the COVID-19 pandemic as elective surgery was suspended. Also, the absence of routine administration of NMB should be considered as an advantage, since it potentially influences BIS values as shown in a study on healthy volunteers. Finally, we prespecified three time points for the evaluation of the surgical conditions to standardize our data irrespective of the operative times. This trial also comes with some limitations. A major limitation is the use of a subjective scale as the primary outcome. The scale was used as surrogate for the surgical overview: it is not validated, but it has extensively been used in previous studies [3–6]. Moreover, we tried to enhance the validity of the scale reporting the intraabdominal pressure values during the laparoscopy. A second limitation is the presence of missing values in the primary outcome (> 5%) which may have affected our results. We did not perform any multiple imputation or sensitivity analyses to take missing scores into account. Possibly, some missing scores may be explained by the conversion to open surgery (i.e., five patients in the CNTRL group and three in the BIS group).

Finally, we were not able to report the continuous BIS values for both groups due to technical challenges. The lack of these data did not allow us to calculate time-weighted average BIS values which would have been more informative than the discrete BIS values recorded by anesthesia providers at the predefined three time points of surgical evaluation.

## 5. Conclusion

We conclude that the use of BIS does not result in a clinically relevant improvement of surgical overview conditions during gynecological laparoscopies compared to the standard practice, when adult patients are anesthetised using a TIVA protocol with propofol and remifentanil. Further research in a different surgical population, preferably major surgery, is still needed.

## Figures and Tables

**Table 1:** Patient baseline characteristics and perioperative details.

	BIS group (*N* = 80)	CNTRL group (*N* = 74)
Median age (IQR), years	44 (30–51)	39 (30–49)
Median BMI (IQR), kg m^−2^	24.8 (22.1–27.8)	24.1 (21.6–27.3)
< 18.5	2	1
≥ 18.5 and < 25	41	42
≥ 25 and < 30	25	21
≥ 30 and < 35	9	9
≥ 35and < 40	3	1
Ethnicity, no./total no. (%)		
Caucasian	79 (98.8)	72 (97.3)
Asian	1 (1.2)	2 (2.7)
Smoking, no./total no. (%)		
Yes	13 (16.2)	14 (19)
No	67 (83.8)	60 (81)
Alcohol, no./total no. (%)		
< 7 units/week	75 (93.8)	72 (97.3)
7–14 units/week	5 (6.2)	2 (2.7)
Prior abdominal surgery, no./total no. (%)		
No	49 (61.3)	34 (45.9)
Laparoscopy	14 (17.5)	20 (27)
Open surgery	11 (13.7)	15 (20.3)
Both	6 (7.5)	5 (6.8)
Type of surgery, no./total no. (%)		
Salpingectomy or salpingo-oophorectomy	54 (67.5)	56 (75.7)
Hysterectomy	15 (18.7)	11 (14.9)
Diagnostic laparoscopy	11 (13.8)	7 (9.4)
Median duration of surgery (IQR), minutes	50 (35–81)	53 (35–78)
Median IAP (IQR)		
At pneumoperitoneum	12 (12–12)	12 (12–12)
After 10 min	12 (11–12)	12 (12–12)
At pneumoperitoneum deflation	11 (8–12)	10 (8–12)
Conversion to open surgery, no./total no. (%)		
Yes	3 (3.8)	5 (6.8)
No	77 (96.2)	69 (93.2)
Median duration of anesthesia (IQR), minutes	80 (60–110)	85 (65–110)
Median stay in PACU (IQR), minutes	75 (70–90)	80 (68–95)

Abbreviations: BMI, body mass index; IAP, intraabdominal pressure; IQR, interquartile range; PACU, postanesthesia care unit.

**Table 2:** Primary outcome: proportion of excellent surgical field scores.

Excellent surgical field score, no./total no. (%)	BIS group (*N* = 80)	CNTRL group (*N* = 74)	*p* value^a^
At pneumoperitoneum^b^	70/79 (88.6)	64/73 (87.7)	0.86
After 10 minutes^c^	72/74 (97.3)	63/65 (96.9)	0.89
At pneumoperitoneum deflation^d^	69/74 (93.2)	66/66 (100)	0.03

*Note:* The exposure of the surgical field was judged by the surgeon in charge on a four-grade numerical scale: excellent (1), good but not optimal (2), poor but acceptable (3), and unacceptable and impossible to continue the operation (4).

Abbreviations: BIS, bispectral index; CNTRL, control.

^a^A Chi-squared test was used.

^b^1 missing score in the BIS group and 1 missing score in the CNTRL group.

^c^Conversion to open surgery did not allow to grade the surgical conditions in 3 patients of the BIS group and 5 of the CNTRL group. 3 patients had missing scores in the BIS group and 4 patients had missing scores in the CNTRL group.

^d^Conversion to open surgery did not allow to grade the surgical conditions in 3 of the BIS group and 5 of the CNTRL group. 3 patients had missing scores in the BIS group and 3 patients had missing scores in the CNTRL group.

**Table 3:** Secondary outcomes.

	BIS group (*N* = 80)	CNTRL group (*N* = 74)	*p* value
Mean propofol (± SD), mg/kg/hour^a^	6.21 ± 1.39	6.01 ± 1.69	0.41
Mean remifentanil (± SD), μg/kg/min^a^	0.47 ± 0.11	0.42 ± 0.12	0.005
Median fentanyl (IQR), mg^b^	0.15 (0.1–0.2)	0.15 (0.1–0.2)	0.11
Use of mivacurium, no./total no. (%)^c^			
Yes	3/80 (3.8)	5/74 (6.8)	
No	77/80 (96.2)	69/74 (93.2)	0.48
Median OMEs (IQR), mg^b^	10 (0–25)	20 (0–35)	0.13
Nausea, no./total no. (%)^c^			
Yes	15/80 (18.8)	7/74 (8.3)	0.1
No	65/80 (81.2)	67/74 (91.7)	
Vomit, no./total no. (%)^c^			
Yes	0/80 (0)	0/74 (0)	
No	80/80 (100)	74/74 (100)	

Abbreviations: BIS, bispectral index; CNTRL, control group; IQR, interquartile range; OMEs, oral morphine equivalents; SD, standard deviation [12].

^a^ *T*-test.

^b^Wilcoxon rank-sum test.

^c^Chi-squared test.

## Data Availability

The data that support the findings of this study are available on request from the corresponding author. The data are not publicly available due to privacy or ethical restrictions.

## Supporting Information

The supporting file includes the Consort checklist for this study. Additional tables and figures are included to further elaborate data from the study. All additional figures and tables are referenced in the main script. Tables/figures in.
