# Supplementary material for: Bispectral Index and Surgical Space Conditions in Day Surgery Benign Gynecological Laparoscopies: A Double-Blinded Randomized Clinical Trial
Source: Anesthesiol Res Pract. 2025 Feb 23;2025:4558323. doi: 10.1155/anrp/4558323 (PMC11872287; doi:10.1155/anrp/4558323)
Supplement: Supporting Information — Additional supporting information can be found online in the Supporting Information section. [file 4558323.f1.docx]

Bispectral Index and surgical space conditions in day surgery benign gynaecological laparoscopies: a double-blinded randomised clinical trial.

Elena Crescioli^1,2^, Peter Søndergaard Thyrrestrup^1^, Thale Almås^3^

^1^ Department of Anaesthesia and Intensive Care, Aalborg University Hospital, Aalborg

^2^ Department of Clinical Medicine, Aalborg University, Aalborg

^3^ Department of Anaesthesia and Intensive Care, Odense University Hospital, Odense

# Electronic supplementary material

Table of contents

[Electronic supplementary material 1](#_Toc1622716261)

[CONSORT 2010 checklist 2](#_Toc319041776)

[Data source 5](#_Toc1264898348)

[Monitoring in PACU 6](#_Toc1577180349)

[Figure S1: Perioperative BIS values in the BIS group 6](#_Toc709239748)

[Figure S2: Surgical field scores 7](#_Toc314237379)

[Table S1: NRS score in PACU 9](#_Toc212912370)

[Table S2: Primary outcome in the per-protocol population 10](#_Toc996958973)

[Table S3: Secondary outcome in the per-protocol population 11](#_Toc1517816302)

## CONSORT 2010 checklist

| Section/Topic | Item No | Checklist item | Reported on page No |
| --- | --- | --- | --- |
| Title and abstract | | | |
|  | 1a | Identification as a randomised trial in the title | 1 |
|  | 1b | Structured summary of trial design, methods, results, and conclusions (for specific guidance see CONSORT for abstracts) | 2 |
| Introduction | | | |
| Background and objectives | 2a | Scientific background and explanation of rationale | 3 |
|  | 2b | Specific objectives or hypotheses | 3 |
| Methods | | | |
| Trial design | 3a | Description of trial design (such as parallel, factorial) including allocation ratio | 3-4 |
|  | 3b | Important changes to methods after trial commencement (such as eligibility criteria), with reasons | 4 |
| Participants | 4a | Eligibility criteria for participants | 4 |
|  | 4b | Settings and locations where the data were collected | 3 |
| Interventions | 5 | The interventions for each group with sufficient details to allow replication, including how and when they were actually administered | 4-5 |
| Outcomes | 6a | Completely defined pre-specified primary and secondary outcome measures, including how and when they were assessed | 4 |
|  | 6b | Any changes to trial outcomes after the trial commenced, with reasons | No |
| Sample size | 7a | How sample size was determined | 6 |
|  | 7b | When applicable, explanation of any interim analyses and stopping guidelines | No |
| Randomisation: |  |  |  |
| Sequence generation | 8a | Method used to generate the random allocation sequence | 4 |
|  | 8b | Type of randomisation; details of any restriction (such as blocking and block size) | 4 |
| Allocation concealment mechanism | 9 | Mechanism used to implement the random allocation sequence (such as sequentially numbered containers), describing any steps taken to conceal the sequence until interventions were assigned | 4 |
| Implementation | 10 | Who generated the random allocation sequence, who enrolled participants, and who assigned participants to interventions | 4 |
| Blinding | 11a | If done, who was blinded after assignment to interventions (for example, participants, care providers, those assessing outcomes) and how | 5 |
|  | 11b | If relevant, description of the similarity of interventions | 4-5 |
| Statistical methods | 12a | Statistical methods used to compare groups for primary and secondary outcomes | 6 |
|  | 12b | Methods for additional analyses, such as subgroup analyses and adjusted analyses | 6 |
| Results | | | |
| Participant flow (a diagram is strongly recommended) | 13a | For each group, the numbers of participants who were randomly assigned, received intended treatment, and were analysed for the primary outcome | 6 + Fig 1 |
|  | 13b | For each group, losses and exclusions after randomisation, together with reasons | 7 + Fig 1 |
| Recruitment | 14a | Dates defining the periods of recruitment and follow-up | 3 |
|  | 14b | Why the trial ended or was stopped | 6 |
| Baseline data | 15 | A table showing baseline demographic and clinical characteristics for each group | Table 1 |
| Numbers analysed | 16 | For each group, number of participants (denominator) included in each analysis and whether the analysis was by original assigned groups | Fig. 1 |
| Outcomes and estimation | 17a | For each primary and secondary outcome, results for each group, and the estimated effect size and its precision (such as 95% confidence interval) | Fig. 2 + Table 2-5 |
|  | 17b | For binary outcomes, presentation of both absolute and relative effect sizes is recommended |  |
| Ancillary analyses | 18 | Results of any other analyses performed, including subgroup analyses and adjusted analyses, distinguishing pre-specified from exploratory | No |
| Harms | 19 | All important harms or unintended effects in each group (for specific guidance see CONSORT for harms) | Non |
| Discussion | | | |
| Limitations | 20 | Trial limitations, addressing sources of potential bias, imprecision, and, if relevant, multiplicity of analyses | 8 |
| Generalisability | 21 | Generalisability (external validity, applicability) of the trial findings | 7-8 |
| Interpretation | 22 | Interpretation consistent with results, balancing benefits and harms, and considering other relevant evidence | 7-8 |
| Other information | | |  |
| Registration | 23 | Registration number and name of trial registry | 3 |
| Protocol | 24 | Where the full trial protocol can be accessed, if available | 3 |
| Funding | 25 | Sources of funding and other support (such as supply of drugs), role of funders | 9 |

CONSORT: Consolidated Standards of Reporting Trials

## Data source

The following data were collected in both groups: American Society of Anesthesiology (ASA) physical status, ethnicity, prior abdominal surgery (laparoscopic and/or open surgery), age, smoking, alcohol consumption, body mass index (BMI), surgical field scores, amount of anaesthetics, narcotics, neuromuscular block and other adjuvants, BIS values, intraabdominal pressure (IAP), duration of procedure, perioperative complications (i.e. bleeding, conversion to open surgery), numeric rating scale (NRS) and nausea scale in the post-anaesthesia care unit (PACU) assessed at the arrival and every 15 minutes afterwards, all medications given in the PACU, PACU stay, postoperative complications occurred in the PACU needing further evaluation by the surgeon (i.e. bleeding), postoperative complications other than related to the surgery and occurred in the PACU (i.e. respiratory failure, bladder retention).

Data were stored in accordance with the Danish legislation and reported to The Danish Data Protection Agency.

## Monitoring in PACU

The standard monitoring equipment consisted of three lead electrocardiogram (ECG), non-invasive blood pressure and pulse oximetry. Respiratory rate, peripheral saturation of oxygen, heart rate, and ECG were monitored continuously, while airway patency, blood pressure, mental status, neuromuscular function, nausea and vomiting and pain were frequently reassessed, typically every 15 minutes. Patient’s postoperative nausea were recorded using a 3-grade numerical scale; eventual vomiting was also registered. The pain score was assessed using a numeric rank scale (NRS) from 1 to 10.

### **Figure S1: Perioperative BIS values in the BIS group**

**
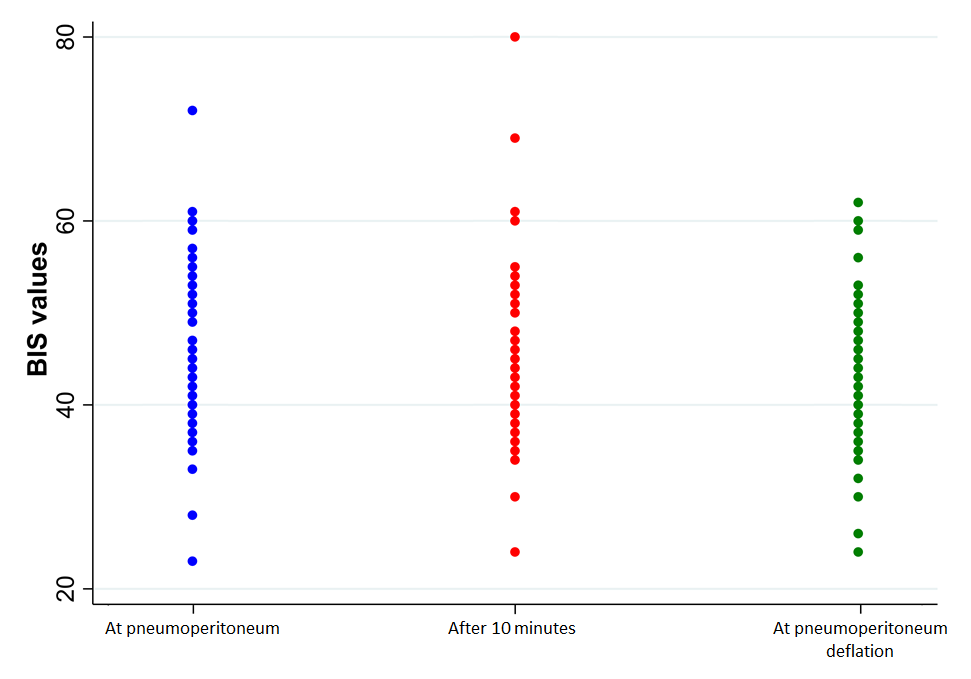
**

**BIS: Bispectral Index**

**BIS values recorded at three time points during surgery.**

### **Figure S2: Surgical field scores**

**
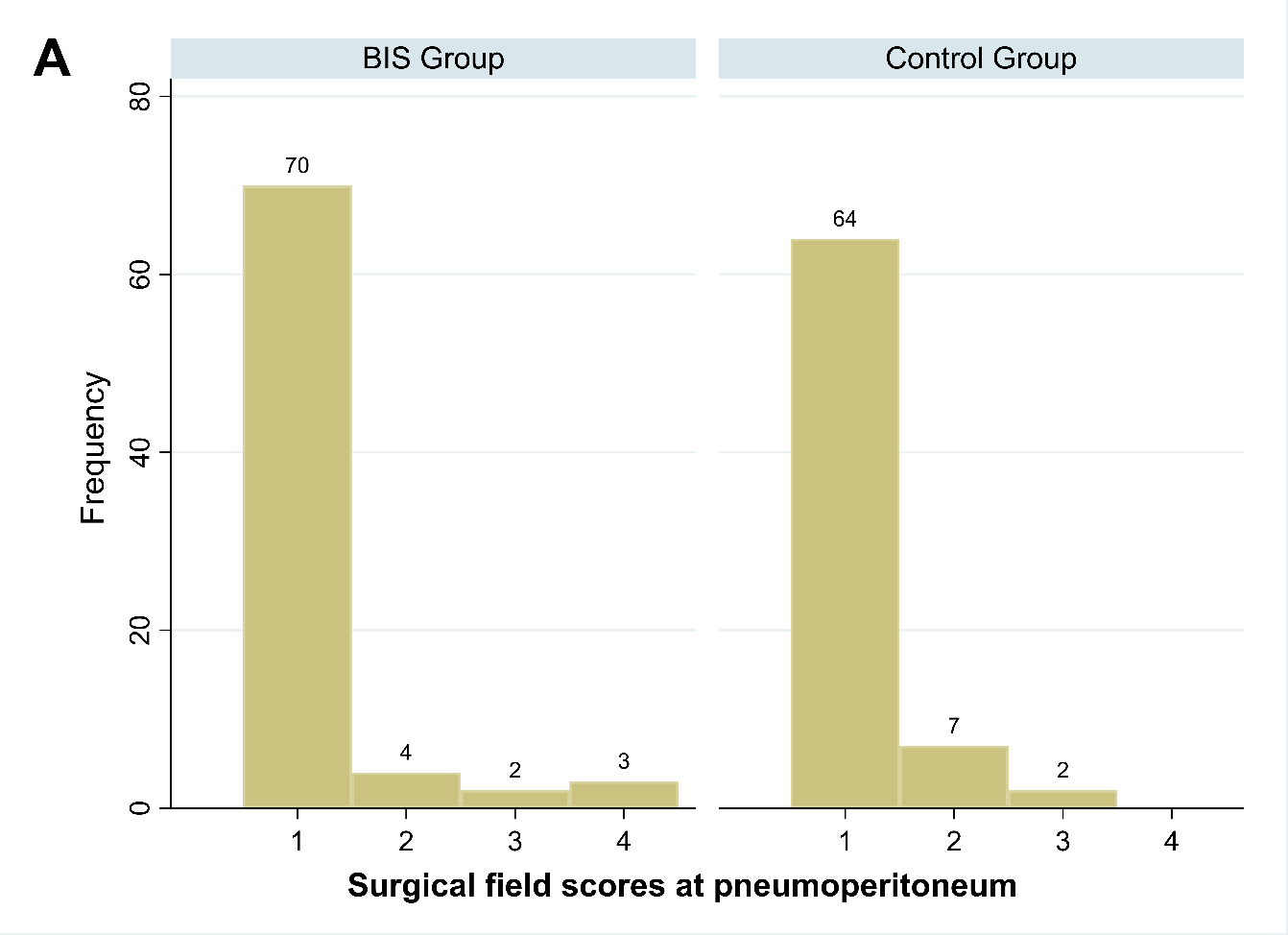
**

**
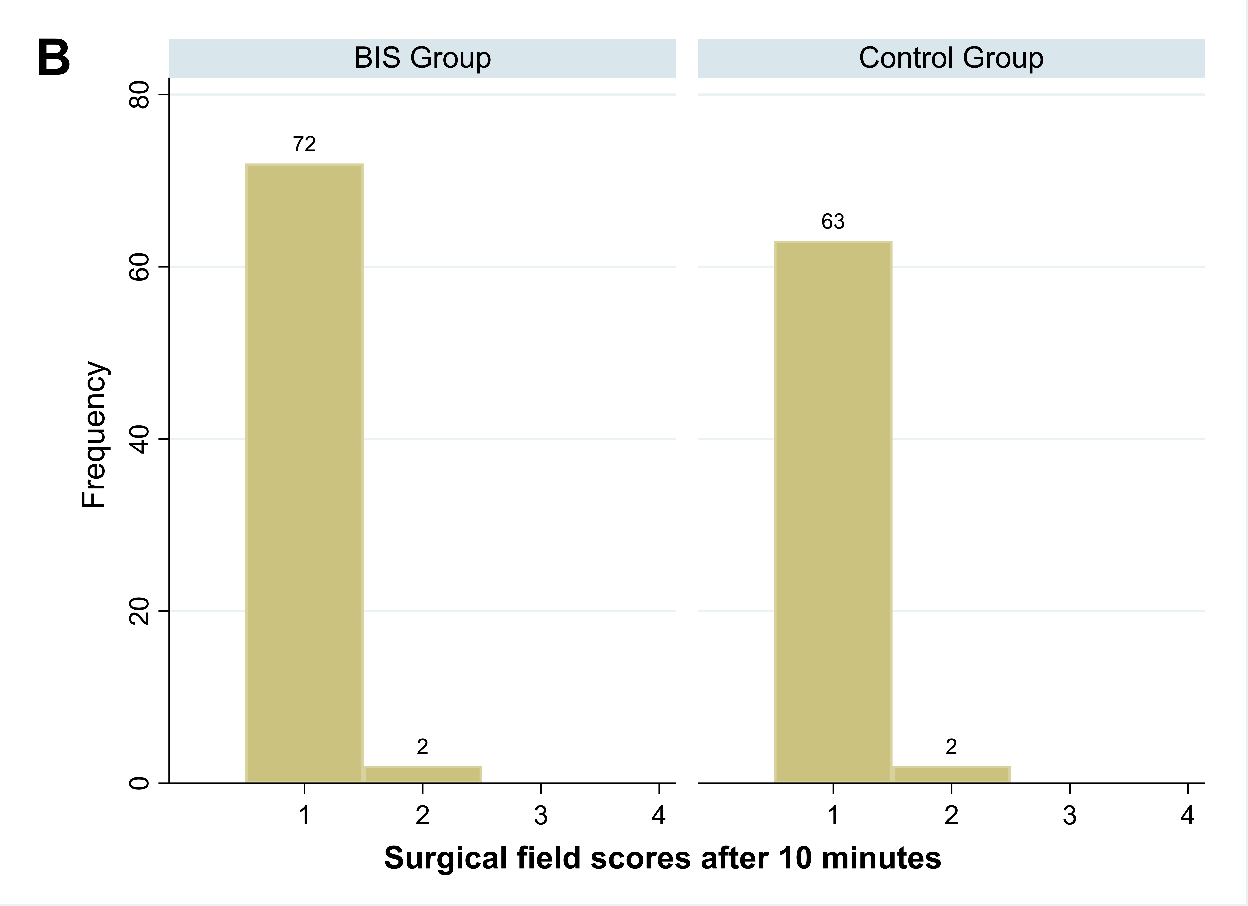
**

**
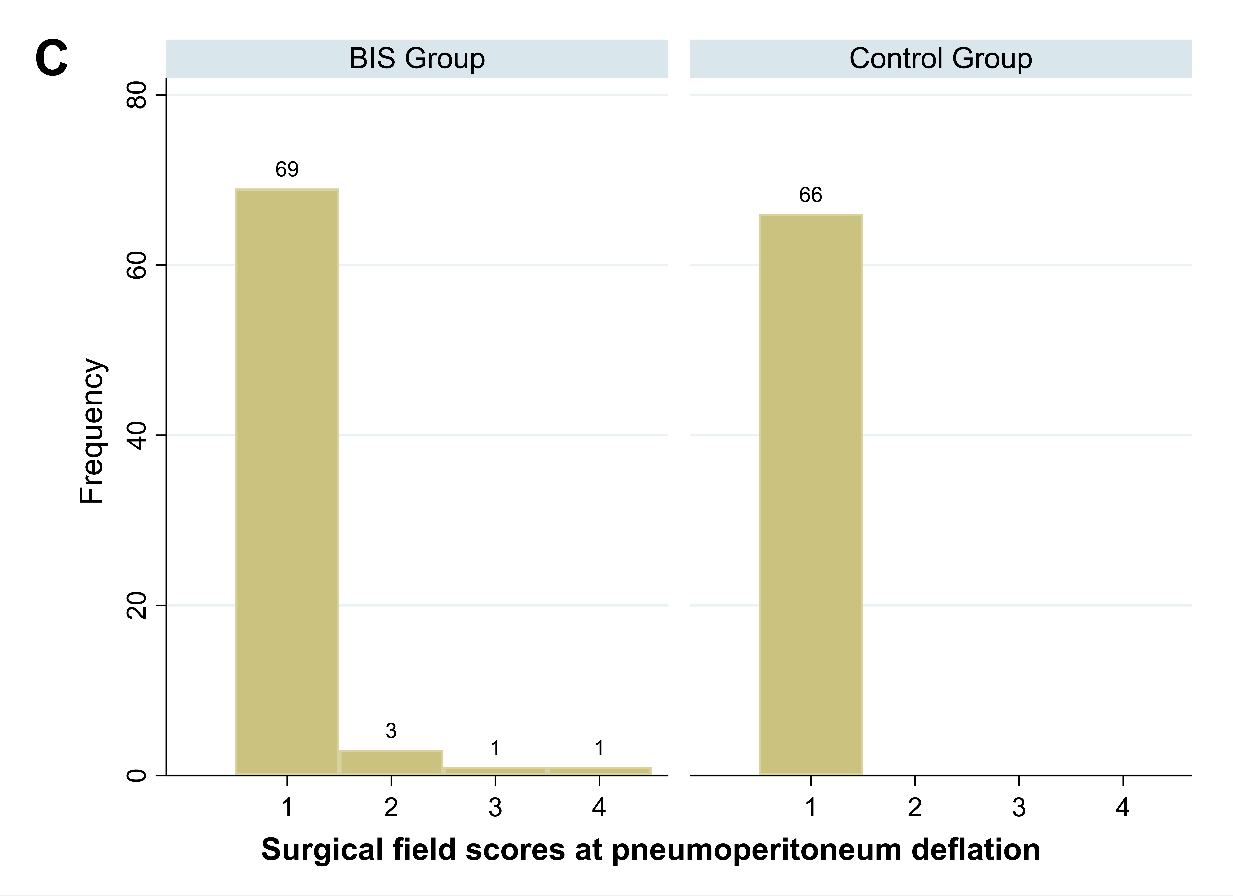
**

**BIS: Bispectral index**

**Surgical field scores are displayed in the BIS group and control group, respectively at start of pneumoperitoneum (A), after 10 minutes (B), and before deflation of the pneumoperitoneum (C).**

### **Table S1: NRS score in PACU**

| NRS in PACU | Group B  (N = 80) | Group C  (N = 74) | P-value^a^ |
| --- | --- | --- | --- |
| NRS at arrival in the PACU, no./total no. (%)  0-4  5-10 | 54/80 (67.5)  26/80 (32.5) | 49/74 (65)  25/74 (35) | 0.87 |
| NRS at 30 minutes, no./total no. (%)  0-4  5-10 | 53/80 (66.3)  27/80 (33.8) | 44/74 (58)  30/74 (40.5) | 0.38 |
| NRS at discharge from PACU, no./total (%)  0-4  5-10 | 75/80 (93.8)  5/80 (6.3) | 73/74 (98.7)  1/74 (1.3) | 0.12 |

NRS: numeric rating scale; PACU: post-anaesthesia care unit.

### **Table S2: Primary outcome in the per-protocol population**

| Excellent surgical field score, no./total no. (%) | Group B  (N = 80) | Group C  (N = 72) | P-value |
| --- | --- | --- | --- |
| At pneumoperitoneum | 70/79 (88.6) | 62/71(87.3) | 0.81 |
| After 10 minutes | 72/74 (97.3) | 61/63 (96.8) | 0.87 |
| At pneumoperitoneum deflation | 69/74 (93.2) | 64/64 (100) | 0.03 |

Primary outcome reported as the proportion as excellent surgical field scores. The exposure of the surgical field was judged by the surgeon in charge on a four-grade numerical scale: excellent (1); good but not optimal (2); poor but acceptable (3); unacceptable and impossible to continue the operation (4).

### **Table S3: Secondary outcome in the per-protocol population**

|  | Group B  (N = 80) | Group C  (N = 72) | P-value |
| --- | --- | --- | --- |
| Mean propofol (± SD), mg/kg/hour^a^ | 6.21 ± 1.39 | 6.01 ± 1.71 | 0.43 |
| Mean remifentanil (± SD), μg/kg/min^a^ | 0.47 ± 0.11 | 0.42 ± 0.12 | 0.005 |
| Median fentanyl (IQR), mg^b^ | 0.15 [0.1 to 0.2] | 0.15 [0.1 to 0.2] | 0.12 |
| Use of Mivacurium, no./total no. (%)^c^  Yes  No | 3/80 (3.8)  77/80 (96.2) | 5/72 (6.9)  67/72 (93.1) | 0.48 |
| Median OMEs (IQR), mg^b^ | 10 (0 to 25) | 22.5 (0 to 35) | 0.11 |
| Nausea, no./total no. (%)^c^  Yes  No | 15/80 (18.8)  65/80 (81.2) | 6/72 (8.3)  66/72 (91.7) | 0.06 |
| Vomit, no./total no. (%)^c^  Yes  No | 0/80 (0)  80/80 (100) | 0/72 (0)  72/72 (100) |  |

SD: standard deviation; IQR: interquartile range; OMEs: oral morphine equivalents.

^a^T-test

^b^Wilcoxon Rank-Sum test

^c^Chi-squared test
